# Active Inference and Cooperative Communication: An Ecological Alternative to the Alignment View

**DOI:** 10.3389/fpsyg.2021.708780

**Published:** 2021-08-12

**Authors:** Rémi Tison, Pierre Poirier

**Affiliations:** Department of Philosophy, Université du Québec à Montréal (UQAM), Montreal, QC, Canada

**Keywords:** active inference, communication, language, cooperative communication, ecological psychology, affordance

## Abstract

We present and contrast two accounts of cooperative communication, both based on Active Inference, a framework that unifies biological and cognitive processes. The mental alignment account, defended in Vasil et al., takes the function of cooperative communication to be the alignment of the interlocutor's mental states, and cooperative communicative behavior to be driven by an evolutionarily selected adaptive prior belief favoring the selection of action policies that promote such an alignment. We argue that the mental alignment account should be rejected because it neglects the action-oriented nature of cooperative communication, which skews its view of the dynamics of communicative interaction. We introduce our own conception of cooperative communication, inspired by a more radical ecological interpretation of the active inference framework. Cooperative communication, on our ecological conception, serves to guide and constrain the dynamics of the cooperative interaction via the construction and restructuring of shared fields of affordances, in order to reach the local goals of the joint actions in which episodes of cooperative communication are embedded. We argue that our ecological conception provides a better theoretical standpoint to account for the action-oriented nature of cooperative communication in the active inference framework.

## Introduction

In this paper, we critique an account of cooperative communication recently proposed by Vasil et al. (2020) situated in the active inference framework. We then present our own account of cooperative communication, based on an alternative ecological interpretation of the active inference framework. In recent years, two broad philosophical and theoretical interpretations of the active inference framework have emerged. The active inference framework is a unified theory of biological and cognitive processes in theoretical biology and neuroscience (Friston, 2010, 2012). The first interpretation of active inference is internalist and emphasizes traditional, intentionalist psychological constructs (the traditional cognitive science ontology). It views priors as beliefs, the downward flow of signals as inference (perception or action), and the upward flow as decision making based on the feedback given by prediction error, and the goal of the system as reducing prediction error, and thus minimizing free energy. The main example of this view is Hohwy (2013), but it is also present in many of Friston's writings and can be viewed here and there in Clark (2016). The second is externalist and emphasizes ecological, relational constructs. Anderson (2014) promotes this new ontology, but some of it has been used for a few generations now by ecological psychologists to understand perception and action (Gibson, 1979). The relational ontology of ecological psychology was developed as an alternative interpretation for the active inference framework by enactivist-minded ecological psychologists (Bruineberg and Rietveld, 2014; Bruineberg et al., 2018a); see also (Ramstead et al., 2020). The ecological interpretation of the active inference framework is centered on the ecological concept of affordance and emphasizes sensorimotor dynamical interaction. It tends to downplay the representationalist interpretation of neural dynamics, speaking instead of self-generated and externally generated signals (Aitchison and Lengyel, 2017) and their cancellation or modulation (amplification or attenuation), causal intermediaries (Orlandi, 2016), and optimal grips on fields of affordances as the metastable dynamical states that minimize free energy (Bruineberg and Rietveld, 2014).

Friston and Frith (2015a,b) see also (Friston et al., 2020) apply the first broad interpretation of active inference to the question of nature and mechanisms of communication, and Vasil et al. (2020) is a rich extension of Friston and Frith's original account, rooted in the work of Tomassello on the biological and cultural evolution of language. They aim to account for the type of cooperative communication which they take to be characteristic of human communication. Following Tomasello, they take cooperative communication to be a species-specific type of human behavior that has the function of aligning mental states with other individuals^1^. On their active inference account, cooperative communication is the principal means for humans to gather evidence for the adaptive prior belief that their mental states are similar to those of other humans in their ecological niche. Theirs, we believe, is an important contemporary addition to our understanding of communication, using elements of the active inference framework to integrate a wide variety of aspects of communication into a coherent framework. We believe, however, that a more thorough-going ecological interpretation of the active inference framework can provide a more fruitful account of communication. This new account avoids the shortcomings of Vasil et al.'s account (identified in section Problems for the alignment view) by depicting cooperative communication as a way to directly coordinate behavior in contexts of joint action (Tison and Poirier, forthcoming; Clark, 1996; Gauker, 2003; Fowler et al., 2008; Galantucci, 2009; Tylén et al., 2010; Fusaroli et al., 2014a; Di Paolo et al., 2018). In this paper, we will describe Vasil et al. (2020) account of communication (section Cooperative communication as mental state alignment) and then evaluate it from an ecological perspective (section Problems for the alignment view). Finally, we introduce the main tenets of the ecological interpretation of active inference to sketch our own, ecological, account of communication (section The ecological account of communication).

## Cooperative Communication as Mental State Alignment

In what follows, we present Vasil et al.'s active inference account of cooperative communication. We cannot do justice here to the richness and breadth of Vasil et al.'s proposal and its wide theoretical implications. We will therefore stick to a review of those key points that are relevant for the purposes of this article.

Vasil et al. aim to account for cooperative communication on the basis of the active inference framework (Friston and Stephan, 2007; Friston, 2010; Friston et al., 2017), which is a formal integrated theory of brain function and biological organization (Friston, 2012, 2013). Active inference subsumes the various processes by which biological systems manage to maintain their organization (i.e., survive) under a unified theoretical postulate, the free energy principle.

The starting point of the free energy principle is the observation that biological systems are systems that have a statistical tendency to find themselves in a particular subset of all the possible states available to them. This subset corresponds to the set of states compatible with their survival, that is, the set of states in which they can maintain their organization. By virtue of their structure, biological systems therefore determine a probability distribution over the range of their physically possible states, with a higher probability assigned to states compatible with their survival. States incompatible with their survival have what is called high surprisal, which is a measure of the degree to which such states are unexpected given this probability distribution. To survive, biological systems must minimize surprisal, which entails that they must strive to find themselves in states that have a low surprisal for them. However, given that they don't have a direct access to surprisal, they instead minimize a quantity to which they do have access and that is postulated to constitute an upper bound on surprisal: free energy. Given that free energy is an upper bound on surprisal, minimizing free energy automatically minimizes surprisal.

Organisms keep track of their free energy by embodying a generative model, generating sensory predictions concerning the state of the organism. Minimizing free energy is the process of reducing the discrepancy between what the generative model predicts and the sensory input to which these predictions are compared^2^. This discrepancy can be reduced either by revising the generative model's predictions (perceptual inference) or by changing the sensory input so that it matches the generative model's predictions (active inference). By continually adjusting their generative model to the statistical properties of the sensory input coming from their ecological niche, as well as transforming their ecological niche so that sensory input corresponds to their generative models' predictions, organisms become attuned to their ecological niche in such a way that statistical properties of the niche can be predicted from the generative model, and vice-versa (Bruineberg et al., 2018b; Constant et al., 2018).

Organisms act in their ecological niche according to action policies, which are constituted of prior beliefs, instantiated in the generative model as probability distributions over sensory states, that specify hierarchically organized sequences of action^3^. At any given time, the action policy pursued by an organism is the action policy that is expected to reduce the most free energy for the organism (Friston et al., 2015; Pezzulo et al., 2018). In the active inference framework, behavior is therefore driven by these prior beliefs, understood as probability distributions in the higher levels of the generative model constraining and contextualizing predictions at lower levels.

The free energy expected under action policies can be factored into two elements: epistemic value, which generates actions used to gather information about the statistical properties of the niche, and pragmatic value, which generates actions used to act in the niche to produce outcomes predicted by the generative model. Action policies that have a high epistemic value are considered to be salient for the organism (Parr and Friston, 2017), and, leading to a better grasp on the statistical regularities of its niche, they allow the generative model to devise more efficient pragmatic action policies.

The salience associated with epistemic value is to be distinguished from the precision of predictions, which encode the degree to which a prediction error at a given level of the hierarchical generative model will affect and update predictions at higher levels. Salience associated with epistemic value is a property of action policies that actively sample the niche to learn statistical regularities, while precision is a measure of the confidence of the generative model in these sensory samplings. They both account for different elements of what is generally considered to be the psychological construct of “attention,” but must be kept distinct in the active inference framework.

Finally, some of the prior beliefs guiding action policies are taken to be “adaptive priors” (Badcock et al., 2019a,b), which are “evolutionarily endowed, heritable beliefs that guide characteristic patterns of cognition and behavior in conspecifics” (Vasil et al., 2020, p. 2). These adaptive priors, transmitted genetically, epigenetically or culturally, constrain the set of action policies that will be instantiated in the generative model to favor action policies that will optimize free energy minimization.

The active inference framework has been extended in recent years to account for various social and cultural phenomena. In particular, social niches are taken to contain cultural affordances relying on shared regimes of attention (Ramstead et al., 2016), and deontic cues (Constant et al., 2018) automatically generating action policies taken by social agents in the niche to reliably lead to free energy reduction. These conceptual additions provide useful resources to account for social phenomena such as social conformity (Constant et al., 2019), narratives (Bouizegarene et al., forthcoming), scripts (Albarracin et al., 2021), and social cognition (Veissière et al., 2019).

Vasil et al.'s contribution belongs to this line of work attempting to extend the active inference framework to various social explanatory targets. They aim to produce an active inference account to explain the cooperative communication that is characteristic of human communicative behavior. Following Tomasello's work on the subject (Tomasello et al., 2005; Tomasello, 2008, 2014), they adopt the view that the function of cooperative communication is to align the mental states of the communicating individuals, which they take as their conceptual starting point and explanatory target. On this view, humans engage in communicative behavior in order to produce the result that they have similar mental states to their conspecifics^4^.

On Tomasello's view, cooperative communication is generated by an “evolutionary selected” (Vasil et al., 2020, p. 3) *mutual expectation of cooperativeness*, posited to have been fixed in the human cognitive architecture by ancestral selective pressures such as obligate cooperative foraging to serve as a motivation for cooperative communication. It is composed of a cognitive component (the ability to share mental states with others) as well as a motivational component (the motivation to share mental states with others) (Tomasello et al., 2005). The mutual expectation of cooperativeness hypothesis is justified by evolutionary game theory and the interpretation of extant primates' and preschool children's behavior. Vasil et al. suggest that mental state alignment, for instance alignment of attentional states, intentions, goals, etc, is crucial for successful cooperation and coordination (Skyrms, 2001; Tomasello, 2014). There would therefore have been strong selection pressures for motivations to align mental states with conspecifics in the context of obligate cooperative foraging of our evolutionary past. The importance of mental state alignment in human communication is also supported by studies in infant development, notably the important work of Tomasello on joint attention (Liszkowski et al., 2007; Tomasello et al., 2007). Experimental work by Tomasello et al. has shown that infants become irritated when adults ignore their communicative acts (Liszkowski et al., 2004). Joint attention allows individuals to ground punctual acts of communication by attending to the same referent, on the basis of which a “common ground,” that is, a “set of mental states (knowledge, beliefs, emotions, etc.) that is inferred to be reliably shared with others” (Vasil et al., 2020, p. 4) is posited to develop.

Vasil et al. suggest that the active inference framework provides the conceptual resources to explain cooperative communication understood as mental state alignment. Their key proposal is the idea that humans' generative models predict that their mental states are similar to the mental states of the other humans sharing their ecological niche. Vasil et al. postulate that humans' generative models contain a particular type of adaptive prior predicting that their mental states are aligned with those of their conspecifics: “natural selection has endowed humans with an adaptive prior for alignment; i.e., an adaptive prior preference for action policies that generate sensory evidence that reliably indicates that their own mental states are aligned with, or similar to, those of conspecifics” (Vasil et al., 2020, p. 2). Adaptive priors, recall, are “evolutionarily endowed, heritable beliefs that guide characteristic patterns of cognition and behavior in conspecifics” (Vasil et al., 2020, p. 2; see also Badcock et al., 2019a,b). This adaptive prior for alignment is taken to be one of the elements (the “motivational” component) constituting the mutual expectation of cooperativeness (Vasil et al., 2020, p. 5). It will bias the selection of action policies toward policies that produce sensory outcomes reliably indicating that the communicating individuals' generative model is aligned with the generative model of those around them. Typically, it will lead to the selection of action policies disambiguating the mental states of others and producing mental state alignment. This allows the alignment prior to constrain the individual's action-perception cycles toward the “unsurprising” result of mental state alignment, in turn minimizing free energy relative to this prior belief. Communicative behavior is therefore cast as an evidence gathering process for the alignment prior belief.

An interesting aspect of Vasil et al.'s proposal is the idea that the adaptive prior for alignment and the process of generative models alignment it generates plays out at multiple nested temporal scales. At the timescale of interaction, the alignment prior will generate action policies leading interacting individuals into coupled action-perception cycles in which they attempt to align as well as disambiguate each other's mental states, in order to confirm the success of the alignment. This will among other things produce proximate motivations for communication such as declarative motivations to align the mental states of the receptor to those of the producer, and interrogative motivations to explore the niche, including the part of the niche constituted by the mental states of others. The alignment prior also entails that communicating individuals will attempt to optimize the relevance of their acts of communication, where relevance roughly means the trade-off between the complexity of processing the act of communication and the quantity of information transmitted through this act (Sperber and Wilson, 1995). For speakers inferred to have significantly divergent mental states, policies generating more complex acts of communication will become salient because they will allow them to align themselves more efficiently, whereas speakers already having a significant common ground will adopt policies leveraging this common ground to produce simpler acts of communication to align mental states.

The alignment prior can also help us to understand the dynamics of communication at an ontogenetic timescale. Being embedded in an ecological niche comprizing already enculturated individuals and their stereotyped and culturally stabilized behavior, developing individuals will learn regimes of expectations (Ramstead et al., 2016) and deontic cues (Veissière et al., 2019) indicating salient and culturally relevant action policies by aligning themselves to those individuals. This will produce a process of asymmetric enculturation (Renzi et al., 2017) where the developing individual tends to align itself to the enculturated and stable individual more than the other way around. Moreover, continually engaging in coupled action-perception cycles leading toward alignment allows developing individuals to learn spatiotemporally deeper sets of action policies regulating their communicative behavior. Vasil et al. suggest that various elements of language learning, such as grammar (Perfors et al., 2011) and word learning (Yildiz et al., 2013), can be explained in this way.

Finally, Vasil et al.'s proposal allows us to model the dynamics of the evolution of communicative systems at the timescale of cultural evolution (glossogeny). The process by which communicative systems, defined as sets of form-meaning pairings, evolve can also be understood as a process of alignment. Communicative systems will tend to minimize their own free energy and stabilize themselves in the particular subset of their complete state space which optimizes the trade-off between simplicity and expressibility introduced above. Basic communicative systems composed of simple and scarcely informative acts of communication such as pointing gestures will evolve toward more complex, hierarchically deeper and increasingly arbitrary communicative systems (Tamariz and Kirby, 2016). The communicative constructions composing the communicative system, determined by hierarchically deeper action policies, thus become more expressive while limiting the complexity of learning and using these constructions.

Vasil et al. suggest in short that human cooperative communication is generated by the adaptive prior for alignment, which affects the dynamics of communication at these multiple nested temporal scales. The alignment prior constrains the selection of action policies toward action policies providing evidence that the generative models of communicating individuals are aligned, i.e., that they have similar mental states. In the next section, we will present some problems for this view of the function of communicative behavior.

## Problems for the Alignment View

As we saw in section Cooperative communication as mental state alignment, the main driver of cooperative communication on Vasil et al.'s (2020) account is the prior belief of a speaker that its mental states are aligned with those of its interlocutor. Alignment at many levels between interlocutors is indeed an important and well-studied aspect of communication. It has been found that interlocutors tend to imitate the syntactic structures of each other's utterances (Pickering and Branigan, 1999; Branigan et al., 2000; Gries, 2005) as well as their lexical choices (Brennan and Clark, 1996; Orsucci et al., 2006). Interlocutors also tend to align many other components of language in the course of a conversation, including accent and speech rates (Giles et al., 1991), phonetic properties such as pitch and loudness (Lelong and Bailly, 2011; Pardo et al., 2017) and overall behavior (Louwerse et al., 2012). Moreover, these alignments seem to entrain each other, so that alignment at one level facilitates alignment at other levels (Branigan et al., 2000; Cleland and Pickering, 2003). These “interactive linguistic alignments” (Pickering and Garrod, 2004) are among others hypothesized to have the function of easing the heavy cognitive burden of engaging in complex linguistic interaction (Pickering and Garrod, 2004, 2013; Garrod and Pickering, 2009; Dale et al., 2014). On Pickering and Garrod's view, they typically culminate in higher level alignments of cognitive processes and situation models enabling mutual understanding and social coordination. Partly inspired by these results, Vasil et al. (2020) adopt the view reviewed above that (1) the function of cooperative communication is the alignment of the mental states of the interlocutors (the alignment view), and (2) that cooperative communicative behavior is explained by the instantiation in their generative models of an adaptive prior belief that their mental states are so aligned (the alignment prior).

Although we recognize that interactive alignment is an important aspect of communication, we are reluctant to adopt these two theses. In what follows, we will review various problems associated with the first thesis. In short, we believe that the alignment view (1) overemphasizes the role of alignment in communication, (2) is unable to account for an important type of communicative act in cooperative communication, (3) fails to account for the role of the pragmatic context in determining the manner and degree of alignment, (4) fails to recognize the pragmatic nature of relevance. A final worry, which we won't develop here into a full argument, is that the alignment view problematically introduces a strong discontinuity between cooperative and non-cooperative communication. We discuss these in turn below. As the second thesis provides an explanation for the conception of communication exposed in the first thesis, abandoning this conception of communication renders the second thesis obsolete: if cooperative communication does not have the function to align mental states, there is no need to postulate an adaptive prior to explain this function. Finally, we will argue in section The ecological account of communication that these problems would be solved following the adoption of (1) an ecological interpretation of active inference and of (2) a conception of the function of cooperative communication which is in keeping with this ecological interpretation; that is, which puts action at the center stage of cooperative communication.

### The Problem of Complementary Joint Actions

The first problem comes from recognizing that in many cases of communicative interaction in cooperative contexts, good coordination will come from the interlocutors explicitly *not* having the same mental states. That is because although many joint actions benefit from synchronized behavior of its participants, which requires them to do more or less the same thing at the same time and implies that they indeed have at least similar mental states, many other joint actions rather require complementary behavior (Dale et al., 2014; Fusaroli et al., 2014b), which implies that they entertain different sensorimotor predictions. As Fusaroli et al. put it: “It is often by doing, thinking and saying different things that interlocutors achieve what an individual alone would not, and it is aligning on specific things, not indiscriminately, that does the job” (2013, p. 149). Tomasello himself seems to recognize a similar point when he says: “overall, then, collaborative activities require both an alignment of self with other in order to form the shared goal, and also a differentiation of self from other in order to understand and coordinate the differing but complementary roles in the joint intention” (Tomasello et al., 2005, p. 681)^5^.

If we move a couch together, for example, we have to synchronize our behaviors and lift it at the same time, which putatively requires that we have the similar sensorimotor prediction that we will lift the couch at time *x*. However, once we start moving the couch, one of us should make sensorimotor predictions corresponding with forward walking movement while the other should make sensorimotor predictions corresponding to backward moving movement. Thus, it seems that many acts of cooperative communication in cases of joint action requiring complementary behavior will be aimed at producing behavior or instill mental states in an interlocutor which are different from the speaker's behavior or mental states. In the active inference framework, this implies that these acts of communication serve to induce predictions that are different from those of the speaker.

Vasil et al. could reply that the deep hierarchical structure of the generative model allows that alignment at higher levels produces differences at lower levels. Indeed, two agents could align themselves on an action plan which would determine differing lower level action policies depending on the role that is assigned to them in this action plan (e.g., we align ourselves on the action plan that we move the couch in a given direction, which implies that you walk forward and I walk backward). In this case, acts of communication in the context of such a complementary action can still be understood as producing mental states alignment concerning the action plan of this complementary action. However, once such an action plan is established, it is to be expected that agents will also produce acts of communication directly aiming at the coordination and regulation of the differing lower level action policies, which will not necessarily aim to produce mental state alignment (e.g., “go forward, not backward”; see section The problem of imperative acts of communication). This seems to show that in at least some cases, the function of cooperative communication is not the alignment of mental states. As we see it, the mental alignment view overemphasizes alignment: alignment is taken as primary and differences as contingent, whereas both should be functions of the joint action that the act of communication serves.

### The Problem of Imperative Acts of Communication

Secondly, although Vasil et al.'s proposition can account for informative and interrogative acts of language, which serve, respectively, to align a hearer's mental states with those of the speaker and vice-versa, the role of imperative acts of language in their account remains a mystery. Imperative acts of language, uttered by a speaker in order to produce a determinate behavior on the hearer's part, do not aim to align the mental states of the interlocutors. Rather, they are uttered to produce determinate effects in the context of the interaction. This is an important problem given that imperatives are frequent in contexts of cooperative communication and cannot be discarded as marginal (Clark, 1996; Aikhenvald, 2010).

One might nevertheless try to depict imperative acts of language as aiming toward mental state alignment by stating that imperatives aim to elicit in the hearer a prediction with the same content than the prediction that generated the act of communication of the speaker. For instance, we could interpret a toddler's asking for milk (and having the prediction that he has milk) as aiming to produce in its caregiver the prediction that the toddler has milk, which would then count as an instance of mental state alignment given that the toddler has a prediction with the same content. However, this solution doesn't quite work. Intuitively, the conditions of satisfaction of an imperative are not fulfilled until the predicted state of affair obtains. If a mother tells her son to tie his shoes, she won't be satisfied with him just wanting to tie his shoes. If for a reason or another he wants to do it but is unable to, rather than taking the goal of her imperative to be achieved, she will opt to do it herself to produce the outcome that she could not obtain with the act of communication. It seems likely that one cannot affect the behavior of an agent without affecting its mental states, but this does not mean that acts aiming to affect behavior are also acts aiming to affect mental states. As the previous example shows, if an imperative instills the proper mental state in the hearer but, for various possible reasons, does not elicit the proper behavior, the goal pursued by the speaker in producing this act of communication has not been met. Moreover, for whatever reason, a speaker could produce an act of communication while intending that a hearer desires to do something without intending her actually doing it. Under the current suggestion, it seems that this distinction would be lost. In the end, speakers utter imperative acts of language to produce effects in the world (more specifically, in the behavior of hearers), not to produce mental states in hearers^6^.

This seems to show that at least some acts of cooperative communication do not aim at the alignment of mental states^7^. The problem of cooperative communication in complementary joint action reviewed above seems to derive from, or is at least related to the problem of imperative acts of language. Indeed, acts of communication produced to coordinate complementary behavior will often be imperative acts of language^8^.

### The Problem of the Pragmatic Modulation of Alignment

The third problem consists in the fact that the degree and manner in which interlocutors align themselves must be modulated in function of parameters external to the simple imperative of aligning mental states, namely, in function of the context of the joint action being performed. It is known that various levels of linguistic alignment in situations of cooperative communicative interaction must be modulated in function of the pragmatic context and the nature of the joint action being executed to produce successful coordination. For example, in a joint task, automatic and indiscriminate alignment of lexical choices diminish collaborative performance, while context-sensitive alignment of task-related vocabulary increase performance (Fusaroli and Tylén, 2012; Fusaroli et al., 2012). This shows that blind and inflexible linguistic alignment is not necessarily beneficial to cooperative interaction, whereas linguistic alignment functionally oriented toward the joint goals of the interaction is always preferable. We submit that the same is true for the alignment of mental states. Selective and targeted alignment of mental states relevant to the local goals of the joint action and the evolving context of the interaction will always be preferable to a continual and automatic process of alignment of mental states, which could be detrimental to cooperative interactions [see for example Coco et al. (2018), who shows that gaze alignment can decrease performance in a collaborative task].

Vasil et al. seem to be aware that the alignment process must in some way be constrained by such pragmatic parameters, specifying for instance that individuals' communicative action policies must become “sufficiently similar; that is, not identical, but usable” (Vasil et al., 2020, p. 16), and speaking of the necessity “to align mental states to a degree adequate to enable cooperative behavior” (2020, p. 4)^9^. The problem is that nothing in Vasil et al.'s account allows us to determine what amount of alignment is sufficient to attain “usability” or “enable cooperative behavior” in any given communicative interaction, or even how to determine what “usable” means in this context. Neither does Vasil et al.'s account currently offer the resources to explain how the alignment processes governing cooperative communication can be modulated by such factors. Moreover, the pragmatic context determines not just the adequate quantity of alignment, but also what mental states are to be aligned, as we will see shortly. As it stands, Vasil et al.'s proposition does not provide the means to explain these phenomena, given that the end of communicative interaction remains the alignment of mental states, whatever they may be.

Vasil et al. could improve their account in this respect by suggesting that this pragmatic modulation of the mental state alignment could be realized by the modulation of the precision of the communicative action policies, so that, for example, action policies aligning mental states are salient up until mental state alignment is no longer relevant in the context^10^. This would bring their account closer to our own, and we would certainly welcome such an improvement. However, it must be noted that this theoretical addition is not entailed by the alignment prior, which provides no criteria for determining when mental state alignment is relevant and when it is not. An external criterion, independent of the alignment prior, is therefore needed to explain this crucial property of cooperative communication. In a pragmatist account such as our own, this criterion is provided by the local goals of the joint action in which the communicative interaction is embedded, as we will see in section The ecological account of communication.

### The Problem of Contextual Relevance

Finally, the last problem concerns the question of relevance. This problem consists in the fact that Vasil et al.'s account cannot account for the pragmatic nature of relevance, that is, the way in which what act of communication is to be considered as relevant depends on the context of the joint action in which the communicative interaction is embedded. Vasil et al. propose that their account entails Sperber and Wilson's principle of relevance (Sperber and Wilson, 1995), which states, roughly, that speakers will tend to optimize a trade-off between the quantity of information transmitted by their utterances and the cost of processing these utterances for their interlocutors. Indeed, if interlocutors entertain shared expectations concerning the language they speak as well as states of the world (i.e., they have a significant “common ground” [Stalnaker, 1978; Clark, 1996]), they can produce simple utterances transmitting a lot of information and more easily align their mental states to a sufficient degree. On the other hand, if interlocutors have generally divergent expectations, their utterances will have to be much more structurally specific to transmit the same amount of information (Winters et al., 2018). Aligning mental states is thus a way to approximate optimal relevance. However, following Grice's maxim of relation (Grice, 1975), which roughly states that an act of communication must be relevant to the conversation, it seems clear that the relevance of an utterance must also be evaluated in function of the context of utterance^11^. In other words, optimizing relevance means that speakers will also tend to produce acts of communication that are not just relevant in general in the sense of optimizing the quantity of information/cost of processing trade-off independently of any context, but relevant for the interlocutors at a particular moment in the context of the interaction (Clark, 1996; Roberts, 2012).

That an act of communication be relevant in the context of the interaction is a pragmatic principle of discourse at least as important or perhaps even superseding the optimization of the trade-off between the quantity of information and the cost of processing. The maxim of relation is indeed the first Gricean maxim acquired in the development of communicative behavior (Eskritt et al., 2008, Okanda et al., 2015) and is a central element of our daily communicative interactions. This is quite intuitive. Suppose that we are preparing a meal together. Among all the acts of communication that we could produce in this context, many would be optimizing the trade-off but would be completely irrelevant to our preparing the meal. Suppose that I have a choice between producing an optimized but irrelevant utterance “it will rain tomorrow” and a less optimized but relevant “When you need it, you will find a bag of flour in the beautifully carved wooden pantry” (an optimal *and* relevant utterance in this context would be “the flour is in the pantry”), it is much more likely that I will chose the relevant act of communication over the optimized one^12^. Speakers will typically rule out such optimized but irrelevant acts of communication because they strive to produce acts of communication that are relevant at a definite moment in the context of the interaction, even over acts of communication that would more optimally align their mental states. The alignment view cannot by itself account for this contextual relevance principle because it states that communicative interactions aim to maximize the alignment of mental states in general rather than coordinate behavior in function of the pragmatic context and the interaction goals.

Another way to put this point is to say that the alignment view holds that the function of communication is to align mental states, but it doesn't allow us to determine which of their mental states interlocutors will preferably align in determinate interactive contexts. This is because, in its current state, it fails to recognize that communicative interactions are embedded in contexts of joint actions aimed at shared goals and toward which the dynamics of the interaction are oriented. As with the third problem identified above, Vasil et al. could improve their account by putting the active inference's precision-weighting mechanism to work. They could state that a higher precision is to be allocated to the action policies leading to the alignment of the mental states that are considered relevant in the context, to the detriment of the action policies aligning irrelevant mental states. Once again, this would bring their account closer to our own and, in our view, would constitute a significant improvement. However, as stated earlier, the alignment prior does not provide the resources to explain what “relevant in the context” means here. It does not entail the Gricean maxim of relation, and might even conflict it in some concrete cases. This is why the alignment view wrongly predicts that, in the “toy” communicative context introduced above, the action policy of uttering “it will rain tomorrow” will be prefered to potentially less optimized but more relevant communicative action policies, such as uttering “when you need it, you will find a bag of flour in the beautifully carved wooden pantry.” An additional element is needed to account for contextual relevance, which is a central property of our communicative interactions.

Although we won't argue in detail for it here, we suspect that it generally won't be possible to identify what is relevant to a communicative interaction independently of the goals of the joint action being pursued in the interaction (see Clark, 1996). This seems to show that a correct account of the contextual principle of relevance minimally requires a pragmatist conception of cooperative communication, according to which acts of cooperative communication are actions made in view of reaching the local goals of a joint action, and only incidentally aim at aligning mental states.

A final potential source of worry for Vasil et al.'s account, which we won't develop here but is nevertheless worth pointing out, is that their conception of communication seems to entail a strong discontinuity between cooperative communication, taken to be characteristic of human communication, and non-cooperative communication, taken to be characteristic of animal communication. Traditional anthropological accounts of human culture often posit a strong discontinuity between animal cognition and culture, on the one hand, and human cognition and culture, on the other. Often this takes the form of a “cognitive Rubicon” (Donald, 1991; Mithen, 1999), some fundamental cognitive evolution that took place some time before modern Homo sapiens made their appearance that was causally responsible for the appearance of the archeological record associated with human culture, and which is at the core of contemporary culture (though greatly amplified by the cultural evolution of practices and artifacts). In a similar spirit, Vasil et al. state that humans have an adaptive prior for alignment while animals do not, thus implying a strong discontinuity between human and animal communication. Such a saltationist view, also echoed in various places in Tomasello's work, is not necessarily a defect in itself, but for two theories with equal explanatory power, surely a gradualist theory is to be preferred over a saltationist one for the sake of evolutionary continuity.

In the next section, we will briefly introduce our ecological interpretation of the active inference framework before developing a pragmatist conception of cooperative communication based on this ecological interpretation which can overcome the problems reviewed in this section.

## The Ecological Account of Communication

### The Ecological Interpretation of Active Inference

As mentioned in the introduction, there are two main interpretations of the active inference framework currently on offer. The first interpretation aligns itself with traditional cognitive science and states that the process of free energy minimization is the process by which an organism infers the causal structure of the world hidden behind its sensory states (Hohwy, 2013, 2016). In this traditional interpretation, the generative model is to be understood as a structural representation recapitulating the spatiotemporal regularities of the environment (Gladziejewski, 2016; Williams and Colling, 2017; Kiefer and Hohwy, 2018; Williams, 2018).

Against this interpretation, some have recently advocated for an interpretation of the active inference framework more in keeping with the main theoretical tenets of enactive, embodied, and ecological approaches to cognitive processes (Bruineberg and Rietveld, 2014; Bruineberg et al., 2018a; Ramstead et al., 2020). According to this “ecological” interpretation^13^, the ultimate aim of the free energy minimizing agent is not to infer the causal structure of the environment, but rather to maintain its organization in its environmental niche. The generative model is therefore not a structural representation of the causal structure of the environment. It is rather a control system regulating the exchanges of the organism with its environment, in effect “[steering] its interactions (over multiple timescales) with its environment in such a way that a robust brain-body-environment system is maintained” (Bruineberg et al., 2018a, p. 2440).

In the ecological interpretation, the agent minimizes free energy by flexibly engaging with the affordances provided by its environmental niche. Affordances are possibilities for action that the environment supplies (affords) to those organisms that can perform the afforded action (Gibson, 1979; Chemero, 2003, 2009). For a given organism, the set of affordances supplied by its (local, global) environment at a given time is determined by the content of its (local, global) environment at that time, as well as its body configuration, physiology and skills at that time. The spatiotemporally structured set of affordances available at any given moment to an organism is called the organism's *affordance landscape* (Rietveld and Kiverstein, 2014). Some affordances in the affordance landscape of an organism will solicit action more than others. The affordances that solicit action for an organism are perceived as salient by the organism in what is called its *field of affordances* (Bruineberg and Rietveld, 2014; Kiverstein et al., 2019). While the landscape of affordances is the structured set of affordances available to an organism at a given time, the field of affordance is a landscape of affordances weighted by salience. The more an action or a sensorimotor loop reduces free energy for an organism, the more its associated affordance appears as salient in its field of affordances (Friston et al., 2012).

Fields of affordances can usefully be conceived in the terms of dynamical systems theory as fields of attractors determining the behavioral trajectory of organisms. At any given moment, the behavior of an organism results from a competition between the various solicitations present in the organism's field of affordances (Cisek, 2007; Cisek and Kalaska, 2010; Pezzulo and Cisek, 2016). An organism navigating its field of affordances thereby aims to have an “optimal grip” on its field of affordance; it tends to engage in a flexible manner with the solicitations presented in its field of affordance while being selectively open to other affordances in its field so as to continually minimize its free energy (Bruineberg and Rietveld, 2014; Bruineberg et al., 2018a).

It must be stressed that the traditional interpretation and the ecological interpretation of the active inference framework certainly do not exhaust every theoretical possibility. As we see it, they constitute two positions in a constellation of possible interpretations, in which a variety of theoretical positions can be proposed. For instance, the interpretation on which Vasil et al.'s proposal relies seems to stand somewhere between those two interpretations, acknowledging the nature of the generative model as a control system and seemingly not straightforwardly embracing the representationalist commitments of the traditional interpretation, while also not giving the ecological notion of a field of affordance the central role it usually plays in more thorough-going ecological interpretations, relying instead on the more traditional notion of a mental state conceived as a hidden internal state (Vasil et al., 2020, p. 6). We believe that an interpretation of active inference more decisively skewed toward the “ecological position” provides a better framework to account for cooperative communication. In the next section, we sketch an account of cooperative communication based on such an interpretation.

### The Pragmatist Conception of Cooperative Communication in Ecological Active Inference

We will now briefly present the main elements of our conception of cooperative communication under this ecological interpretation of active inference (for a fuller presentation, see Tison and Poirier, forthcoming), before explaining how it manages to avoid the problems faced by Vasil et al.'s account. Following our pragmatist view, communication in general has to be understood as a form of action. In this view, communication does not have the function of entertaining representations of the world and transmitting these representations to others. It is rather a way of doing things in the world to further particular goals. In the active inference framework, action is active inference; that is, the modification of the incoming sensory stimuli so that it matches the predictions of the organism's generative model, thus minimizing free energy. The particularity of *communicative* active inference is that it minimizes free energy not by acting directly on the world, but rather by affecting the behavior of other organisms: An organism A produces an act of communication C to a target organism (or organisms) B (or B', B”, etc.) when it produces an action in order to change B's field of affordance so as to make B act (select an action in its field of affordance) in a way that minimizes A's expected free energy (Tison and Poirier, forthcoming). In our ecological view, communicating organisms affect the behavior of their target by producing signals in various modalities that modify the field of affordances of the target, which will constrain the target's behavior in particular ways leading to free energy minimization in the communicating organism (see Borghi et al., 2013; van den Herik, 2018 for similar propositions, though not formulated in the active inference framework). Understood in this way, communication is a form of *socially extended active inference*: the sensorimotor control loops regulating an organism's internal states extend in the world to harness the behavior of other organisms (see Fotopoulou and Tsakiris, 2017; Fusaroli et al., 2014a for the similar idea of dialogically extended mind).

Cooperative communication results from the application of this pragmatist conception of communication to contexts of joint action. The pragmatist approach to *cooperative* communication states that cooperative communication is always embedded in a joint action or a cooperative activity pursued by the interlocutors, and that the function of cooperative communication is first and foremost to coordinate the behavior and the interaction of these interlocutors toward the reach of the goals of this joint action. Successfully achieving a joint action in turn reduces the free energy of its participants. Such joint actions can be as varied as buying something (from somebody), playing a game, performing a ritual, etc. Once the communicative interaction and the relevant social practices are established in a community, joint actions having explicitly communicative goals become available, such as telling a story, explaining a scientific theory, exchanging political views, etc.

As noted by Vasil et al. it seems clear that cooperative communication emerged from situations of collaboration, theoretically illustrated by scenarios such as the stag hunt game (Skyrms, 2001), where participants choose to renounce a low risk and low reward individualistic prize for a high risk and high reward shared prize. But whereas Vasil et al. suggest that cooperative communication serves to align mental states, which would in turn help coordination in collaborative situations, we propose that cooperative communication serves to directly regulate and constrain the joint activity in the collaborative situation (Fowler et al., 2008; Fusaroli et al., 2014b; Di Paolo et al., 2018). An important advantage of this view is that the only priors required for cooperative communication are those required for engaging in a joint action (see Blomberg, 2016a,b for a deflationist account), rendering unnecessary the additional mental alignment prior postulated by Vasil et al. Moreover, as we will see, communicative interaction understood in this way naturally leads to the alignment of the generative models of the interactants at ontogenetic and cultural evolutionary (glossogenetic) timescales, without the need to postulate an additional adaptive prior driving this alignment process.

In our account, joint actions take place in contexts of action, or pragmatic contexts, which are spatiotemporally structured sets of elements of the environment that are relevant in light of the local goals of the joint action^14^. The environment of a joint action presents various elements, situations, and events at various timescales that are more or less relevant in function of the current stage of the joint action, and successfully undertaking a joint action requires skillful engagement with the right elements of the context at the right time. Suppose once again that we are preparing a meal, which at a given point requires the use of flour. At this point in our joint action, the flour becomes an element that is relevant in the context of action. Succeeding in our making the meal depends at that moment on our performing correctly the proper action on this element of the context.

The participants in a joint action navigate fields of affordances comprising regular environmental and cultural affordances but also the affordances of interacting in various ways with other participants in the joint action (Kono, 2009; Worgan and Moore, 2010), the other participant's own affordances (Creem-Regehr et al., 2013; Maranesi et al., 2014; Borghi, 2018), and collective affordances (Weichold and Thonhauser, 2020) specifying joint action possibilities. Given the common goals instituted by their participation in the joint action, participants will often perceive the same affordances as salient, while also recognizing that these affordances are considered salient by the other participants in the joint action, thus creating shared sollicitations, or shared relevant affordances (Kiverstein and Rietveld, forthcoming)^15^. Sets of shared affordances constitute what we call shared fields of affordances. Shared fields of affordances are spatiotemporally structured sets of affordances jointly salient for the participants in an interaction (see Krueger, 2011 for the similar notion of “we-space”). The affordances that stand out in the shared field are the affordances that are of shared relevance for the joint action in which the participants are engaged.

The optimal reach of the joint action's goals typically requires that the shared field of affordances corresponds to the context of action: the affordances that are salient and solicit action at a given time in the shared field of affordances must correspond to the elements of the environment that are relevant at the same time for the joint action^16^. To return to our example, the success of our joint action of making the meal requires that the flour, which becomes at some point a relevant element of the context of action, solicits the proper action at the right time in our shared field of affordances. The shared field of affordances can effectively be conceived as instantiating the participants' common take on the context of action. We can consider that what is part of the shared field of affordances is part of the common ground between the participants.

In our ecological view, acts of cooperative communication serve to construct and actively manage shared fields of affordances and perform various kinds of moves in the context of action in order to optimally reach the goals of the joint action. By their communicative behavior, interlocutors attempt to manage the shared field of affordances so that it continuously corresponds to the evolving context of action, to ensure that what is relevant to their project at any given time is salient for them at that time. Producing the utterance “the flour is in the pantry” while we are preparing a meal, for example, is therefore understood as a way of locating in our shared field the affordance of using the flour, so that we can act on this affordance at some point in our joint action.

Communicative behavior so understood allows participants to functionally constrain the dynamics of the interaction and organize their behavior toward the successful completion of the joint action (Tison and Poirier, forthcoming; Verbrugge, 1985; Raczaszek-Leonardi and Scott Kelso, 2008; Fusaroli et al., 2014a; Raczaszek-Leonardi, 2016; van den Herik, 2018). As an individual organism can have an “optimal grip” on its field of affordances (Bruineberg and Rietveld, 2014), allowing it to selectively and skillfully engage with its solicitations to continuously minimize free energy, participants in a joint action can have an optimal grip on the shared field of affordances. Here too, all else being equal, the participants' having an optimal grip on their shared field of affordances will lead to free energy minimization, because it will typically allow them to optimally achieve the joint action in which they are engaged^17^. Acts of cooperative communication contribute to the participants maintaining an optimal grip together on the shared field of affordances by functionally constraining their behavior through active management of their field of affordances.

Dialogue has long been recognized in discourse analysis as the navigation of shared hierarchical structures variously called context spaces (Reichman, 1978), focus spaces (Grosz and Sidner, 1986), or topics (Brown and Yule, 1983) in order to accomplish joint projects (Clark, 1996; Bangerter and Clark, 2003). We suggest that shared (nested) fields of affordances can play the role of such hierarchical structures traditionally used to explain various properties of communicative interactions. For instance, Bangerter and Clark (2003) suggest that utterances such as “uh-huh,” “yeah,” “okay,” or “all right” serve to mark various types of transitions in the hierarchical structure of joint actions. “Uh-huh” and “yeah” mark the horizontal transition between steps of a joint action, while “okay” and “all-right” mark the (vertical) exit of a subproject in the joint action. Such utterances can serve to manage and coordinate the joint action, but also the communicative interaction itself, which is a part of the joint action. In our framework, these utterances can be seen as marking horizontal and vertical transitions between the affordances of the hierarchically structured shared field of affordances that are taken to be relevant at that particular point.

Some acts of communication are specifically designed to construct the shared field of affordances which the participants will take as the context of their action. Informative acts of language, traditionally conceived as being uttered with the intention that the hearer acquires a belief, are used to point out or indicate the presence of an affordance which is relevant to the joint action. The utterance “the flour is in the pantry” mentioned earlier is an act of communication of this type. In this view, informative communication is something like “a technique for pointing” (Baggs, 2015, p. 260) affordances. Informative acts selectively activate some of the affordances in the landscape of affordances that are relevant, or will be relevant, at a given point in the context of action.

As individuals become skillful at manipulating fields of affordance, the context of action may come to comprise or even be exclusively constituted of elements that are absent from the immediate spatiotemporal context, beyond the “here-and-now” of the conversation (Knott, 2012), for example when the goal of a joint action is to plan a future joint action or discuss past events (e.g., there is no flour in the kitchen and we have to go buy some in a nearby store). In these cases, informative acts of language are used to construct shared simulations or *reenactments* (Kiverstein and Rietveld, 2018) of fields of affordances that the participants can use as situation models to coordinate their behavior in relation to the spatiotemporally distant elements that are relevant to their projects. Simulations of fields of affordances can be entertained in hierarchical generative models by decreasing the precision of sensorimotor predictions in the lower levels of the model to activate and maintain predictions of fields of affordances in higher levels despite their incongruence with current sensory input. This could allow generative models to simulate fields of affordances associated with the interaction with a spatiotemporally distant element (e.g., the store), even if current sensory input indicates the absence of this element (we are still in the kitchen), because the precision of this sensory input would be decreased, which would diminish its influence on the higher levels of the hierarchy. These higher-level simulations of fields of affordances adaptively constrain the interaction with current fields of affordances, thereby producing a *hierarchical affordance competition* (Pezzulo and Cisek, 2016), allowing coordination with spatiotemporally distant affordances relevant for the joint action. Here too, communicative acts will have the function of constructing and managing these shared simulations of fields of affordances to further the goals of the joint action (e.g., “there is a store on the corner, the flour is in the third row”).

In a way, it seems that informative acts of language could be described as having the function of aligning the mental states of the participants, as Vasil et al. suggest, because they have the function of aligning the participants' individual fields of affordances to create a shared field of affordances. However, here, the ultimate goal of the construction of the shared field of affordance is not the alignment itself, but rather the coordination of the interacting individual's behavior in function of determinate pragmatic goals. Interlocutors will therefore always strive to construct fields in which salience is attributed to elements of the context that are relevant at that particular moment in the joint action, rather than simply align fields of affordances. Moreover, it must be noted that, in our proposal, what is aligned is not primarily the generative models themselves, but rather the fields of affordances, which are composed of patterns of salience over affordances partly constituted by structures of the environment (as well as simulations or reenactments of such affordances), which are significantly different from what mental states are usually considered to be. It would therefore be incorrect, or at least misleading, to depict the construction of shared fields of affordances as the alignment of mental states. In contexts where communicative practices are well-established in a given community, the alignment of mental states might itself become the goal of some joint action (e.g., explicit teaching, etc.). These communicative interactions might at this point be correctly described as targeting generative model alignment. However, the resulting communicative interaction will have such a target only because it has been instituted as a common goal of the interaction, and not because it is the function of cooperative communication in general.

The fact that cooperative communication serves the coordination of joint action rather than mental states alignment produces the problem noted above for Vasil et al.'s account that many acts of language won't serve the purpose of aligning mental states (or, in the context of our proposal, aligning fields of affordances), but rather that of performing various moves in the context of action to help the progress of the joint action. Imperative acts of language, for example, play such a role. They serve to act on the context of action through the behavior of the target of the imperative act in a way that is expected to favor the reach of the joint action's goals. Imperatives manage to elicit the proper behavior from their target by highlighting an often immediate solicitation that is to be acted upon by the target (e.g., “take the bag of flour that is in the pantry”). While imperative acts are pragmatic actions, used to attain goals or subgoals in the context of action through a target's behavior, interrogative acts are epistemic actions (Friston et al., 2015; Pezzulo et al., 2018), analog to visual saccades and exploratory behavior, used to reduce uncertainty, explore and sample the context of action through the informative acts produced by an interlocutor in response to the interrogative act (e.g., producing “where is the flour” instead of exploring the kitchen to find it by yourself). Imperative acts of language can therefore be cast as driven by action policies with high pragmatic value, whereas interrogative acts of language are driven by action policies with high epistemic value.

To summarize, the function of cooperative communication is to constrain the dynamics of the interaction toward the reach of the joint action's goals. Interlocutors do this by producing acts of communication used to construct a shared field of affordances adequate to the context of action and by manipulating and restructuring the constructed field to perform various kinds of moves in this context to optimally achieve the joint action.

### The Problems of the Alignment View in Light of the Ecological Account

This general conception of cooperative communication and its implementation in our ecological interpretation of the active inference framework can overcome the problems of Vasil et al.'s account exposed above (section Problems for the alignment view). These problems come from Vasil et al.'s adoption of the claim that the function of cooperative communication is the alignment of mental states, which tends to downplay or neglect the fundamentally pragmatic nature of communicative interactions. This has for effect that they cannot account for episodes of cooperative communication that do not aim at the alignment of mental states, such as imperative acts of language and various communicative interactions in complementary joint action.

In our ecological view, acts of communication are not only used to construct a shared field of affordances, which is indeed a form of alignment (though not of mental states, as noted above), but also to perform various moves in the context of action to attain the goals of the joint action. This allows us to understand imperative acts and communication in complementary joint action as such moves, serving to constrain the hearer's behavior through a modification of its field of affordances in order to produce determinate effects on the context of action and reach the goals of the joint action.

Furthermore, Vasil et al. seemingly cannot explain how the goals of the joint action being pursued in a communicative interaction determine and orient the communicative behavior of the participants and the dynamics of the interaction. Indeed, indiscriminate linguistic or epistemic alignments will be less efficient than contextual and task-sensitive alignments to optimally reach the goals of the joint action (Fusaroli et al., 2012; Coco et al., 2018). Our proposition implies that the alignment of mental states, which we propose should rather be understood as the construction of a shared field of affordances, will be modulated in function of the goals of the joint action because, in our view, communicative behavior has primarily the function of constraining the dynamics of the interaction toward shared goals. Communicative action policies will be salient for the interlocutors only insofar as they are taken to contribute to the progress of the joint action in which they are involved. The manner and degree of alignment will therefore be determined by the task in which the interlocutors are engaged, not by a context-independent imperative of aligning mental states.

Relatedly, following the contextual relevance principle, speakers will strive to produce utterances that are relevant in the context of the joint action, even over utterances that would otherwise optimize the alignment of mental states. Once again, our proposition predicts this. In our view, it is in the nature of cooperative communication that speakers will try to produce utterances that are relevant with respect to the joint action's goals. Indeed, only affordances relevant for the joint action will appear as salient and worth pointing out by speakers to constitute a shared field of affordances that corresponds to the context of action. Saying “it will rain tomorrow” during a joint action of preparing a meal is not a salient communicative action policy because it does not activate a shared affordance or set of affordances that is taken to be relevant for the joint action. Speakers engaged in cooperative communication will therefore always produce acts of communication to constrain the dynamics of the interaction with respect to elements that are relevant, or taken as relevant, in the context of action^18^. Contrary to Vasil et al.'s account, contextual relevance is built in the nature of what we take cooperative communication to be.

Finally, our proposition eschews the strong discontinuity between cooperative and non-cooperative communication that seems to be entailed by Vasil et al.'s account. In our view, cooperative communication is a particular case of a general conception of communication according to which acts of communication are active inferences that affect the behavior of other organisms through modifications of their field of affordances, and which are produced to reduce the communicating organism's free energy (Tison and Poirier, forthcoming). Organisms engaged in situations of joint action will use acts of communication to manipulate a *shared* field of affordances, in order to constrain each other's behavior toward the reach of the joint action. This implies that cooperative communication is not an entirely different type of communication brought about by a radical and sudden socio-cognitive innovation, the crossing of some Rubicon, but rather involves the use of preexisting communicative capacities in situations of ecological pressure for collaboration (Moore, 2017). This view of communication is more amenable to gradualist explanations of the evolution of cooperative communication and language (Moore, 2017, 2018)^19^.

## Conclusion

In this paper, we presented two conceptions of cooperative communication built on the active inference framework. We argued that the first conception, although not without merits, suffers from some important problems insofar as it tends to downplay or neglect the pragmatic nature of cooperative communication. We proposed that an alternative conception based on an ecological interpretation of the active inference framework provides a more compelling view of cooperative communication, in which the function of acts of communication is not to align mental states *per se*, but rather to constrain the dynamics of the interaction, through the construction and manipulation of shared fields of affordances, toward the goals of the joint action pursued in the interaction.

As mentioned above, we certainly do not deny that an important and recurrent effect of cooperative communication is the alignment of generative models. In fact, we should expect that episodes of cooperative communication will almost systematically result in some form of alignment between interlocutors. When participants in a joint action construct and navigate a shared field of affordances together, they *ipso facto* integrate shared regimes of attention and shared expectations concerning their ecological niche (Ramstead et al., 2016; Constant et al., 2019). Given the learning mechanisms at work in their generative models, individuals interacting with each other will naturally come to have similar expectations concerning their environment as well as concerning each other's behavior. The alignment of the generative models of interlocutors is a normal consequence of their interaction. It is therefore to be expected that repeated interactions will lead to a significant alignment of generative models in linguistic communities on developmental (Roepstorff et al., 2010) and cultural evolutionary timescales (De Boer, 2011).

If we observe the effects of cooperative communication at such timescales, it might thus seem natural to conclude that the function of cooperative communication is indeed the alignment of mental states, especially given how it facilitates in return the coordination of joint action. But we have argued that this view mischaracterizes the dynamics of cooperative communication at the interaction timescale.

Crucially, conversations have goals. Acts of communication are ways of constraining the dynamics of the interaction in order to pursue these goals. To paraphrase Bruineberg et al. (2018a), the communicating brain is not a scientist. Ultimately, interlocutors are not trying to “disambiguate the mental states of inscrutable others” (Vasil et al., 2020, p. 11), but rather to act with others in shared fields of affordances. In communities infused with shared communicative practices, it might become useful to predict that you share expectations with other members of your community in order to expand your common ground, in turn facilitating coordination and communication in that community. However, this prediction is not an evolutionarily selected and inherited adaptive prior, but rather an empirical prior learned from repeated communicative interactions, and, though it is definitely useful for cooperative communication, it is not a necessary condition for it^20^.

In the end, our own account is not that far from Vasil et al.'s proposition, which we see as an important stepping stone toward the formulation of an active inference theory of communication. We share the view that the active inference framework provides us the tools to formulate a theory of cooperative communication embedded in a unified theory of brain function and behavior (Friston, 2009, 2010). Moreover, we agree that alignment is an important element of cooperative communication, and we celebrate their illuminating application of the active inference framework to the study of the dynamics of communicative behavior at developmental and cultural evolutionary timescales. However, we suggest that an ecological interpretation of the active inference framework leads us toward a more plausible conception of cooperative communication at the timescale of the interaction, according to which acts of communication serve to functionally constrain the dynamics of the interaction through the modification of fields of affordances and mental states alignment must rather be interpreted as the construction of shared fields of affordances oriented toward the joint action's goals.

Our proposal is still quite speculative and a lot of empirical and conceptual work remains to be done to properly flesh it out and provide adequate support for it. Some notions will have to be explained in more detail in future work. For instance, the notion of simulations of fields of affordances remains to be fully fleshed out in active inference terms. Moreover, our account, at the moment, primarily targets fairly basic cases of cooperative communication in simple joint actions (e.g., two individuals preparing a meal). It will have to be scaled up to account for more complex cases of linguistic interaction involving abstract concepts (e.g., two individuals discussing the active inference framework). Such communicative interactions and the joint actions in which they are embedded are heavily scaffolded on sociocultural practices and institutions and draw on a variety of epistemic resources, such as conceptual contents, that we cannot introduce here. We aim to provide an active inference account of conceptual contents and their role in communicative interaction in our future work.

Despite these current limitations, we believe that our pragmatist conception of communication, implemented in the ecological interpretation of active inference, provides the right framework to underscore the action-oriented nature of all communicative interactions and capture the function of communication as a free energy minimizing activity.

## Data Availability Statement

The original contributions presented in the study are included in the article/supplementary material, further inquiries can be directed to the corresponding authors.

## Author Contributions

RT redacted most of the manuscript. PP redacted some sections of the manuscript. The main ideas presented in the manuscript are the result of discussions at collaborative work between RT and PP. The manuscript has been reviewed, modified and improved by both RT and PP. All authors contributed to the article and approved the submitted version.

## Conflict of Interest

The authors declare that the research was conducted in the absence of any commercial or financial relationships that could be construed as a potential conflict of interest.

## Publisher's Note

All claims expressed in this article are solely those of the authors and do not necessarily represent those of their affiliated organizations, or those of the publisher, the editors and the reviewers. Any product that may be evaluated in this article, or claim that may be made by its manufacturer, is not guaranteed or endorsed by the publisher.

## Footnotes

^1^There are various forms of animal communication that can also be considered to be cooperative. However, they do not correspond to what Vasil et al. call cooperative communication, which has the function of aligning mental states for its own sake and is thought to be unique to humans (2020, p. 3). The alternative view of cooperative communication that we propose in this paper is not explicitly committed to the view that cooperative communication is unique to humans. However, cooperative communication in our view comes with the capacity to engage in flexible and coordinated joint actions with others. If only humans can engage in such joint actions (which is not clear, see Boesch, 2005; Suchak et al., 2016), then only humans can cooperatively communicate.

^2^We adopt the seemingly representationalist vocabulary of prediction in this general introduction of active inference, as is often done in the field. We assume however that this vocabulary can be rephrased and eliminated in favor of more ecological constructs, as will be shown in section The ecological interpretation of active inference.

^3^As noted by Vasil et al. such prior beliefs are not beliefs in the folk-psychological sense of propositional attitudes. They are rather to be understood as “(subpersonal) bayesian beliefs” (2020, p. 2, note 2), which are updated probability distributions over sensory states.

^4^It is not perfectly clear to us what Vasil et al. mean by “mental states,” which they leave somewhat undefined. “Mental states” seems here to be a catch-all term including behavioral dispositions (p. 13), attentional and perceptual states (p. 3–4), and hidden representational states such as intentions (p. 3) and beliefs (p. 4). We are not sure how to relate some of these psychological constructs to the active inference framework. As noted above, the prior beliefs constituting the generative model are not to be understood as beliefs in the traditional folk-psychological sense, and action policies are also significantly different from what intentions are usually taken to be. In absence of a clearer account, we will also understand “mental states” in this broad sense, and suppose that these have correlates in the predictive machinery of the generative model.

^5^Given this, it is not totally clear that Tomasello would adopt the alignment view as formulated by Vasil et al. (2020).

^6^Even in traditional gricean analyses, the communicative intentions determining the nature of the act of language produced by a speaker are aimed toward the behavior of the interlocutor rather than its mental states in cases of imperative communication (Grice, 1957, p. 384–6; Neale, 1992). In such analyses, an act of language counts as an imperative when it is produced by a speaker intending that the hearer does *x*, and not intending that the hearer desires or intends to do *x*.

^7^Moreover, it could be argued that various other types of use of linguistic communication do not seem to aim at mental state alignment. For instance, linguistic communication can be used for social bonding, entertainment, various types of rituals, etc., which intuitively count as cooperative communication (but might not count as cooperative communication in Vasil et al.'s sense if they *define* it as aiming toward mental states alignment) but do not necessarily aim to elicit similar mental states (see Keiser, 2020).

^8^However, this might not necessarily be so. Consider a situation where a participant A in a complementary joint action with a participant B indicates to B where to find information or instructions relevant for B's complementary action. A might not know or even be interested in knowing this information. It seems that we could possibly describe this situation as a case of an informative act of language used to coordinate complementary behavior and which do not aim at aligning mental states.

^9^This is already a significant improvement from other work on communication in active inference which seem to presuppose that “pure” communication takes place independently of any pragmatic context and for the sole sake of synchronizing generative models (see especially Friston et al., 2020, p. 43–4).

^10^Thanks to an anonymous reviewer for pointing out this possibility.

^11^The only maxim in the category of relation (other categories have two to four maxims) is simply stated by Grice as: “be relevant,” over and above considerations about quantity of information and cost of processing. This laconic and somewhat obscure definition was later clarified by understanding relevance as a function of the goals of the joint action in which the communicative interaction is embedded (see Clark, 1996; Gauker, 2003).

^12^While we are preparing the meal, we could engage in some small talk during which we talk about topics completely unrelated to cooking, such as the weather. In this case, we should say that we are engaged in a supplementary joint action aiming, for example, at the establishment or maintenance of social relations and also in which some optimized acts of communication will be relevant and others will not.

^13^Bruineberg, Rietveld and Kiverstein qualify this interpretation of “ecological-enactive.” However, given the ongoing discussion concerning the compatibility of enactivism and ecological psychology (see e.g., Heft, 2020; Read and Szokolszky, 2020) and the fact that our own presentation of these ideas does not depend on enactivist elements, we prefer to use simply the “ecological” label.

^14^This notion of context is adapted from Gauker (2003, 2011), and is significantly different from more traditional notions of context in pragmatics (e.g., Duranti and Goodwin, 1992; Bergs and Diewald, 2009); see Gauker (1998) on some of these differences).

^15^Shared affordances are distinct from collective affordances. Collective affordances are affordances of joint actions (e.g., the affordance of lifting together a heavy rock). Shared affordances are affordances that are jointly considered to be relevant in the performance of a joint action (e.g., we both consider that using flour is relevant at some point in our joint action of preparing the meal). There are shared affordances that are not collective affordances (using flour could be an action that can be performed alone) and there are collective affordances that are not shared affordances (a participant in a joint action can perceive the liftability of a heavy rock by multiple agents while it is not perceived as such by the other participants, and is therefore not shared).

^16^Strictly speaking, it is not always necessary that the affordances corresponding to relevant elements of the context be shared. A complementary joint action will often not require that each participant perceive each other's affordances of complementary actions. In such cases, it seems that the correspondence between the context of action and the *sum* of the fields of affordances of the participants is sufficient for the success of the joint action. However, the performance of the joint action can often be more flexibly adjusted in the context and more finely coordinated when the complete context of action is well represented in the shared field of affordances (i.e., both participants are aware of what is to be done and what is relevant for the joint action).

^17^In many cases, though, we can expect the generative models of the participants to contain predictions unrelated to the joint action that could generate prediction error even if they maintain an optimal grip on the shared field of affordances. This is just a way of saying that the individual field of affordances and the shared field of affordances are not identical, or in other words that the norms of the individuals are distinct from the norms of the interaction (Di Paolo et al., 2018).

^18^Furthermore, it seems that pragmatist conceptions of communication can provide some additional elements to explain the tendency to optimize the quantity of information/cost of processing trade-off. As a type of action, an act of communication is subject to something like a principle of least action, according to which agents will strive to reach their goals with a minimum of effort. Supposing that the goal of a particular act of communication is to constrain the behavior of the receiver in a particular way, a speaker will tend to produce the act of communication that achieves this result with minimal costs. If the speaker has also learned that there are costs to producing utterances that are too long or hard to process for the receiver (because he will stop paying attention and the intended effect won't be achieved), this principle of least action, applicable for all behavior, implies the optimization of the quantity of information/cost of processing trade-off for communicative behavior in particular.

^19^Moreover, the distinction between cooperative and non-cooperative communication is not only a distinction between human and animal communication, but also a distinction between various types of human communication. Indeed, many important human communicative interactions don't take place in cooperative settings, but rather in strategic or competitive contexts (Saul, 2012; Asher and Lascarides, 2013). We suggest that conceiving these various types of communicative interactions as continuous and as involving the application of the same cognitivo-communicative abilities to different interactive contexts would be more profitable to the development of a general conception of human communication.

^20^To be clear, we are not arguing that it is unnecessary for interlocutors to have some common ground in order for them to engage in cooperative communication, which is hardly plausible on our view given that it seems necessary for joint action. The fact that the interlocutors are engaged in a joint action already entails that they have some common ground: they at least both have the intention to reach the goals of the joint action, and they presumably both perceive at least some common elements of the context of action as salient in their fields of affordances given their shared goals. Rather, we are denying that the *prediction* that interlocutors have common ground is constitutive of cooperative communication, over and above the common ground implied by their joint action.
